# Supplementary material for: Empirical methods for controlling false positives and estimating confidence in ChIP-Seq peaks
Source: BMC Bioinformatics. 2008 Dec 5;9:523. doi: 10.1186/1471-2105-9-523 (PMC2628906; doi:10.1186/1471-2105-9-523)
Supplement: Additional File 1 — A variety of html documents from the USeq web site detailing the available applications, their best usage, output file type descriptions, command line menus, etc. [file 1471-2105-9-523-S1.zip › USeqUserGuides/JavaDoc.zip!/JavaDoc/util/bio/wrappers/BL2SeqHit.html]

BL2SeqHit


|  |  |  |
| --- | --- | --- |
| **Overview** \| **Package** \| **Class** \| **Use** \| **Tree** \| **Deprecated** \| **Index** \| **Help** | |  |
| **PREV CLASS**   **NEXT CLASS** | **FRAMES**    **NO FRAMES**     **All Classes** |  |
| SUMMARY: NESTED \| FIELD \| CONSTR \| METHOD | DETAIL: FIELD \| CONSTR \| METHOD |  |


---


## util.bio.wrappers Class BL2SeqHit

```
java.lang.Object
  util.bio.wrappers.BL2SeqHit
```

---

``` public class BL2SeqHit extends java.lang.Object ```

BL2Seq result container.

---

**Constructor Summary**

`BL2SeqHit()`


| **Method Summary** | |
| --- | --- |
| `int` | `getOrientation()` |
| `int` | `getRawScore()` |
| `java.lang.String` | `getSeq1()` |
| `java.lang.String` | `getSeq2()` |
| `int` | `getStartSeq1()` |
| `int` | `getStartSeq2()` |
| `int` | `getStopSeq1()` |
| `int` | `getStopSeq2()` |
| `void` | `setOrientation(int orientation)` |
| `void` | `setRawScore(int rawScore)` |
| `void` | `setSeq1(java.lang.String seq1)` |
| `void` | `setSeq2(java.lang.String seq2)` |
| `void` | `setStartSeq1(int startSeq1)` |
| `void` | `setStartSeq2(int startSeq2)` |
| `void` | `setStopSeq1(int stopSeq1)` |
| `void` | `setStopSeq2(int stopSeq2)` |

**Methods inherited from class java.lang.Object**

`equals, getClass, hashCode, notify, notifyAll, toString, wait, wait, wait`

**Constructor Detail**

### BL2SeqHit

```
public BL2SeqHit()
```


**Method Detail**

### getOrientation

```
public int getOrientation()
```

---


### setOrientation

```
public void setOrientation(int orientation)
```

---


### getRawScore

```
public int getRawScore()
```

---


### setRawScore

```
public void setRawScore(int rawScore)
```

---


### getSeq1

```
public java.lang.String getSeq1()
```

---


### setSeq1

```
public void setSeq1(java.lang.String seq1)
```

---


### getSeq2

```
public java.lang.String getSeq2()
```

---


### setSeq2

```
public void setSeq2(java.lang.String seq2)
```

---


### getStartSeq1

```
public int getStartSeq1()
```

---


### setStartSeq1

```
public void setStartSeq1(int startSeq1)
```

---


### getStartSeq2

```
public int getStartSeq2()
```

---


### setStartSeq2

```
public void setStartSeq2(int startSeq2)
```

---


### getStopSeq1

```
public int getStopSeq1()
```

---


### setStopSeq1

```
public void setStopSeq1(int stopSeq1)
```

---


### getStopSeq2

```
public int getStopSeq2()
```

---


### setStopSeq2

```
public void setStopSeq2(int stopSeq2)
```


---


|  |  |  |
| --- | --- | --- |
| **Overview** \| **Package** \| **Class** \| **Use** \| **Tree** \| **Deprecated** \| **Index** \| **Help** | |  |
| **PREV CLASS**   **NEXT CLASS** | **FRAMES**    **NO FRAMES**     **All Classes** |  |
| SUMMARY: NESTED \| FIELD \| CONSTR \| METHOD | DETAIL: FIELD \| CONSTR \| METHOD |  |


---
